# Kinetic and Structural Studies of the Plastidial *Solanum tuberosum* Phosphorylase

**DOI:** 10.1021/acsomega.4c06313

**Published:** 2024-09-25

**Authors:** Symeon M. Koulas, Efthimios Kyriakis, Anastasia S. Tsagkarakou, Demetres D. Leonidas

**Affiliations:** Department of Biochemistry & Biotechnology, University of Thessaly, Biopolis 41500, Larissa, Greece

## Abstract

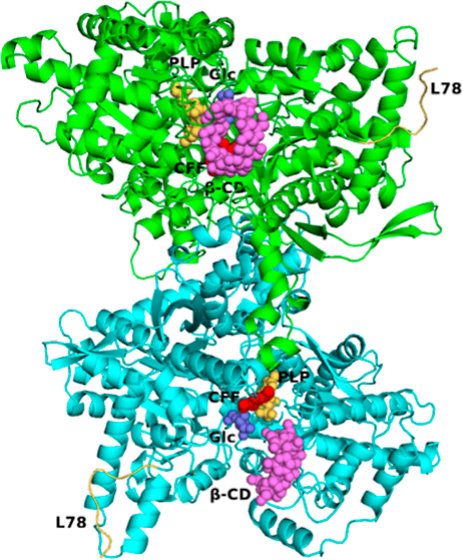

Kinetics and structural studies of the plastidial *Solanum tuberosum* phosphorylase (*st*Pho1) revealed that the most active form of the enzyme (*st*Pho1ΔL78) is composed by two segments generated by proteolytic degradation of an approximately 65-residue-long peptide (L78) approximately in the middle of the *st*Pho1 primary structure. *st*Pho1ΔL78 is 1.5 times more active than the nonproteolyzed enzyme in solution and shows stronger specificity for glycogen, α-d-glucose, caffeine, and β-cyclodextrin than *st*Pho1. The crystal structure of *st*Pho1ΔL78 has been resolved at 2.2 Å resolution and revealed similarities and differences with the mammalian enzymes. The structural fold is conserved as is the active site, while other binding sites such as the inhibitor, the glycogen storage, the quercetin, and the allosteric are not. The binding of α-d-glucose, caffeine, and β-cyclodextrin to *st*Pho1 has been studied by X-ray crystallography and revealed significant differences from those of the mammalian phosphorylases. As *st*Pho1 is capable of catalyzing both starch synthesis and degradation, our studies suggest that the direction of *st*Pho1 activity is regulated by the proteolytic degradation of the L78 peptide.

## Introduction

Glucan phosphorylases share a solid evolutionary relationship among animals, plants, algae, and bacteria. Starch phosphorylase (EC 2.4.1.1, α-d-glucosyl transferase; SP) catalyzes the reversible conversion of starch and inorganic phosphate into glucose-1-phosphate (Glc-1-P), playing an important role in starch metabolism in plants.^1,2^ This enzyme is widely distributed in plant tissues and has been isolated and characterized from *Solanum tuberosum*,^3−5^ green algae *Chlamydomonas reinhardtii*,^6^*Lotus japonicus*,^7^*Hordeum vulgare*,^8^*Arabidopsis thaliana*,^9^*Oryza sativa*,^10^*Spinacia oleracea*,^11^*Pisum sativum*,^12^*Vicia faba*,^13^*Musa acuminata*,^14^ and *Triticum aestivum*.^15^ Higher plants have two distinct types of SP, a plastidial or amyloplastic form (Pho1 or SP-L; low affinity for glycogen) and a cytosolic form (Pho2 or SP-H; high affinity for glycogen).^16−18^ Although these two forms share significant sequence similarity, they largely differ in their molecular sizes, subcellular localization, protein structure, enzymatic kinetic properties, substrate specificities, and expression patterns during plant cell development.^2,18−20^ Pho1 is the major active form of SP since it accounts for more than 95% of the total phosphorylase activity in several higher plants,^10,21−24^ but both forms were found to be active enzymatically.^5,8^

Phosphorylases are enzymes that phosphorolytically degrade polysaccharides. However, as enzymatic catalyzed reactions are reversible, the direction in which they proceed relies on the ratio of substrate to product. Thus, phosphorylase-catalyzed reactions can proceed toward polysaccharide synthesis when the ratio of phosphate/Glc-1-P is much smaller than 2.2.^3^ From early days, it has been suggested that plant phosphorylases, in contrast to animal homologues, catalyze the biosynthesis of starch in plants.^3,25,26^ More recent biochemical and genetic studies in crop plants supported this hypothesis, introducing the idea that starch phosphorylases play a key role in the production and initiation of storage starch.^6,10,27−30^

In mammals, the SP homologue is glycogen phosphorylase (GP).^31^ Biologically active GP is a homodimer that has pyridoxal 5′-phosphate (PLP) as a cofactor, which is regulated allosterically and by phosphorylation. However, this regulation differs for the three different GP isoforms (liver, muscle, and brain).^32,33^ Thus, the liver enzyme (lGP) is regulated by phosphorylation,^34^ the muscle (mGP) responds cooperatively to activation by AMP and to phosphorylation,^35^ and the brain (bGP) responds strongly and noncooperatively to activation by AMP.^36^ GP follows the Monod–Wyman–Changeux model^37^ and exists in two interconvertible GP forms, GPa and GPb. GPa is the Ser14 phosphorylated form with high activity and substrate affinity (R state); GPb is the unphosphorylated form with low activity and substrate affinity (T state).^31^ Apart from the catalytic site in GP, crystallographic studies have identified five additional binding sites (allosteric, new allosteric, inhibitor, quercetin, and glycogen storage).^35^

Phosphorylase sequence alignment for a wide range of species reveals that phosphorylase in higher plants is structurally similar to the animal and bacterial forms (maltodextrin phosphorylase) but differs at the N-terminus.^17,21,38^ The most significant structural difference between the Pho1 and Pho2 forms is a 78-residue-long peptide insertion in the middle of Pho1, termed the L78 segment.^17,39^ The function of this extra peptide varies among species and ranges from the substrate of proteasomes to modulate the degradation of Pho1 in *S. tuberosum* to a nonsignificant effect on biochemical activity in *O. sativa* and *H. vulgare*.^2^

Although various starch phosphorylases are the industrially preferred enzymes for the development of engineered varieties of glucans and starch,^1^ little is known about their biochemical and structural properties, and this is mostly limited to *H. vulgare* Pho1 (*Hv*Pho1),^40^*Ipomoea batatas* Pho1 (*Ib*Pho1),^41^ and *A. thaliana* PHS2.^42,43^ Therefore, the need for more structure–activity relationship data for plant phosphorylases to elucidate their functional mechanism and to develop more biotechnological applications is evident.

*S. tuberosum* phosphorylase is the first starch phosphorylase discovered^3−5^ and has served as a model SP for biochemical studies of plant phosphorylases. Initially, two forms were discovered named “slow” and “fast” isozymes^5^ according to their migration in polyacrylamide-gel electrophoresis. Subsequently, it was found that these were both the same enzyme, *S. tuberosum* Pho1 (*st*Pho1), and the “slow” and “fast” isozymes corresponded to the intact enzyme and to two domains of almost equal molecular weight generated by proteolysis of the intact enzyme, respectively. For *S. tuberosum* L., two Pho1 type phosphorylases have been sequenced.^17,44^ Both proteins are highly similar (81–84%) in their primary structure, except for the N-terminal transit peptide and the large insertion located between the N- and the C-terminal domains. Immunoprecipitation studies^45^ have shown that in tubers, only a homodimer of *st*Pho1 is detectable. The L78 peptide in *st*Pho1 contains a set of negatively charged residues, phosphorylation sites,^18^ and has been reported to sterically hinder large polysaccharides binding.^45^ Excluding the L78 segment, the *st*Pho1 amino acid sequence is 51% and 40% similar to that of the rabbit muscle and *Escherichia coli* phosphorylases, respectively.^17^*st*Pho1 maintains its intact (105 kDa) structure in young tubers but degrades to a 55 kDa form as the tubers mature.^38^

We present here a biochemical analysis of *st*Pho1 together with the crystal structure of *st*Pho1ΔL78, a form of *st*Pho1 that lacks residues 447–510. Of these 64 residues, the first 46 (59%) are part of the L78 segment. The *st*Pho1ΔL78 structure reveals that activation of the enzyme occurs through the proteolytic degradation of the L78 segment and the association of the two remaining protein segments to form the active enzyme. We also studied by kinetics and X-ray crystallography the binding of α-d-glucose, caffeine, and β-cyclodextrin to map the molecular details of their respective binding sites. Our results provide new insights into the functional characteristics and the structure–activity relationship of *st*Pho1. Furthermore, a detailed comparative structural analysis of *st*Pho1 to the mammalian GPs revealed differences and similarities to the various binding sites related to the evolution of this important class of enzymes.

## Materials and Methods

### Protein Purification

**Figure 1:**
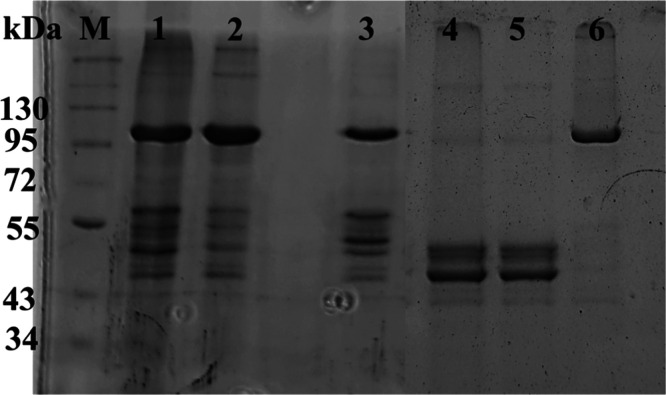
SDS PAGE electrophoresis of the stPho1 samples during proteolysis. Each lane represents stPho1 samples at different stages after the start of incubation. Lane M, protein marker; lanes 1–5, 3, 6, 9, 14, and 28 days since the start of incubation; and lane 6, the stPho1 sample at the start of the incubation.

*st*Pho1 was purified from a natural source by modifying a previously established protocol^46^ and using ion exchange and affinity chromatography. More specifically, 2 kg of *S. tuberosum* mature tubers (50 g, fresh weight; collected from a local farm near Larissa from 4 month old plants and stored no longer than 6 months at 4 °C in the dark) was peeled, sliced, and soaked for 1 h in 3 L of a 0.7% (w/v) Na_2_SO_4_, 0.7% (w/v) sodium citrate solution. Afterward, the slices were washed with distilled water and treated with a juicer. Juice was clarified by centrifugation (10.000*g*, 10 min, 4 °C), and the pH of the clear supernatant was adjusted to 7.2 with 2 M NaOH. The crude extract was brought to 55 °C in a water bath, where it remained for 10 min while stirring. This was flowed by rapid cooling in an ice bath, and the precipitate was removed by centrifugation (10.000*g*, 10 min, 4 °C). Solid (NH_4_)_2_SO_4_ was added to the supernatant solution until it reached a concentration of 60% (w/v) and left overnight at 4 °C. The pellet was collected by centrifugation (10,000*g*, 15 min, 4 °C) and resuspended in a 5 mM sodium citrate (pH 6.3) buffer, containing 1 mM PMSF. Overnight dialysis against the same buffer was followed. After centrifugation (13,000*g*, 25 min, 4 °C), the supernatant was filtered through a 0.45 μΜ filter, and Pho1 was purified using ion exchange (HiTrap Q, GE Healthcare) and affinity chromatography (β-cyclodextrin Sepharose 6B column), using an AKTA purifier. The HiTrap Q column was equilibrated with buffer A (5 mM sodium citrate, pH 6.3). After the sample was loaded, the column was washed with a 5 mM Tris–HCl (pH 7.5) buffer and was eluted with linear gradient concentration of buffer B [5 mM Tris–HCl (pH 7.5), 1 M NaCl]. The active fractions were collected, and the protein was dialyzed against a 25 mM Tris–HCl (pH 7.5) buffer with 0.7 M (NH_4_)_2_SO_4_. The protein was then loaded on a β-cyclodextrin Sepharose 6B column, which was pre-equilibrated in buffer C [25 mM Tris–HCl, pH 7.5, 0.7 M (NH_4_)_2_SO_4_]. The column was washed with buffer C, and the protein was eluted with gradient concentration of buffer D (25 mM Tris–HCl, pH 7.5). This purification procedure resulted in 2.1 mg of pure stPho1 sample per kg of potato tubers based on SDS PAGE. *st*Pho1ΔL78 enzyme was prepared by keeping a solution of *st*Pho1 in 25 mM Tris/HCl (pH 7.5) buffer at room temperature. Enzyme proteolysis was followed by SDS page-electrophoresis, and it was over in 4 weeks (Figure 1).

### Enzyme Kinetics

In vitro, *st*Pho1 can perform the reverse reaction in the presence of higher concentrations of Glc-1-P, where the enzyme adds glucose residues on glucans with direct release of orthophosphate ions, which can then be measured spectrophotometrically using the method of Saheki et al.^47^ Starch granules are composed of two distinct glucose polymers, amylose and amylopectin.^48^ Amylopectin is the major polymer in starch and has α-1,4-linked glucose chains with α-1,6-linked branches every 24–30 glucose units, whereas amylose is composed of long α-1,4-linked linear chains. Kinetic studies were performed using glycogen, whose structure is very similar to that of amylopectin but with more branches (every 8–12 glucose units) due to better solubility. Enzyme assays were performed at 30 °C in the direction of glycogen synthesis using 30 μg/mL of stPho1 or ΔPho1 in a 20 mM sodium citrate buffer (pH 6.0) containing 60 mM KCl, 0.6 mM ethylenediaminetetraacetic acid (EDTA), and 0.6 mM dithiothreitol, using constant concentrations of glycogen (0.2% w/v) and various concentrations of Glc-1-P (3, 4, 6, 10, and 15 mM for *st*Pho1 and 1, 2, 3, 4, 6 mM for *st*Pho1ΔL78) and inhibitors. Initial velocities were calculated from the pseudo-first-order rate constants (k) using the first-order rate equation ([*A*] = [*A*]_0_e^–*kt*^), where [*A*]_0_ and [*A*] are the initial concentration of the sample and the sample’s concentration at various times, respectively, and *t* is the corresponding time (min) following a previously established protocol,^54^ and the nonlinear regression program GraFit^50^ with an explicit value was used for calculating the standard deviation of each point.^49^

### Crystallization, X-ray Data Collection, Structure Determination, and Refinement

*st*Pho1 crystals were grown using the sitting drop vapor diffusion method at 16 °C from drops containing equal volumes of the protein (6 mg/mL) and a buffer composed of 0.1 M Tris–HCl at pH 8.5 and 20% (w/v) PEG 4000. Before crystallization, 1 mM DTT was added to the protein solution. Two different forms of crystals were grown belonging to *P*3_1_21 (unit cell dimensions *a* = *b* = 127.1 Å, *c* = 118.7 Å, α = β = 90°, γ = 120°) and *C*2 (unit cell dimensions *a* = 218.1 Å, *b* = 134.9 Å, *c* = 123.1 Å, α = γ = 90°, β = 91.3°) crystal systems, respectively. To produce crystals of *st*Pho1 in complex with either α-d-glucose, β-cyclodextrin, or caffeine, the protein solution was incubated with 25 mM α-d-glucose, 3 mM β-cyclodextrin, or 7.5 mM caffeine for 20 min on ice before crystallization experiments were set. All crystals of the *st*Pho1-ligand complexes belong to the *C*2 space group with unit cell dimensions very similar to the *C*2 crystal of the free structure. For data collection, crystals were equilibrated in the reservoir solution supplemented with 30% (v/v) glycerol for 30 s, flash-cooled, and stored in liquid nitrogen. X-ray diffraction data were collected using synchrotron radiation at beamline P13 of the EMBL-Hamburg outstation on a Pilatus 6 M detector. Crystal orientation, integration of reflections, interframe scaling, partial reflection summation, and data reduction were performed by the program XDS,^51^ while scaling and merging of intensities was performed by Aimless.^52^ Data resolution limits were determined by the CC^1/2^ criterion.^53^ The native *st*Pho1 structure was solved by molecular replacement with the program MOLREP^54^ within the CCP4 package^52^ using barley endosperm plastidic starch phosphorylase (PDB entry: 5LR8)^8^ as the search model. The refined native structure then served as the initial model for the complex structures. Crystallographic refinement of the complexes was performed by maximum-likelihood methods using REFMAC.^55^ Depending on the number of unique reflections, *B* factor refinement was performed employing either TLS anisotropy restraints (free *st*Pho1 from *P*3_1_2_1_ crystals and β-CD complex) or NCS restraints (free *st*Pho1 from C2 crystals, glucose, and caffeine complexes). Manual model building and map inspection was performed with COOT.^56^ The validity of the refinement procedure was checked using the PDB REDO server.^57^ Hydrogen bonds and van der Waals interactions were calculated with the program CONTACT within the CCP4 package^52^ applying a distance cutoff of 3.35 Å and 4.0 Å, respectively. Figures were prepared with PyMOL.^58^ Data collection and refinement statistics are listed in Table 1.

**Table 1:** Summary of the Diffraction Data Processing and Refinement Statistics for the *st*Pho1ΔL78 Structure and Ligand Complexes^a^

complex			α-D-Glc	β-CD	caffeine
space group	*P*3_1_21	*C*121	*C*121	*C*121	*C*121
asymmetric unit	monomer	trimer	trimer	trimer	trimer
resolution (Å)	2.2 (80.8)	3.3 (123.0)	3.1 (117.1)	2.8 (123.1)	3.7 (49.5)
reflections measured	437,703 (36,762)	157,661 (14,196)	158,753 (13,756)	260,420 (13,413)	112,803 (14,193)
unique reflections (*F* > 0)	53,632 (4611)	52,489 (4606)	54,908 (4608)	87,204 (4507)	37,747 (4635)
*R*_merge_	0.160 (1.221)	0.088 (0.588)	0.191 (1.024)	0.09 (0.818)	0.432 (2.239)
completeness (%)	100 (100)	97.9 (99.4)	95.0 (97.6)	98 (99.4)	98.0 (98.9)
⟨*I* /σ(*I*)⟩	10.4 (2.1)	9.5 (2.5)	4.1 (0.9)	7.4 (1.3)	3.3 (1.2)
redundancy	8.2 (8.0)	3.0 (3.1)	2.9 (2.9)	3.0 (3.0)	3.0 (3.1)
CC^1/2^	0.996 (0.746)	0.997 (0.805)	0.977 (0.349)	0.995 (0.654)	0.947 (0.378)
reflections used for refinement	50,984	49,944	52,296	82,781	35,841
B Wilson (Å^2^)	43.8	84.3	80.6	87.7	81.4
no. of water molecules	323	63	130	100	18
no. of atoms per asymmetric unit	7005	20,082	20,215	20,377	20,046
no. of ligand atoms			36	231	14
*R* (%)	17.6 (35.1)	25.4 (39.2)	23.6 (37.6)	21.8 (37.7)	27.9 (39.2)
*R*_free_ (%)	22.9 (36.9)	30.0 (41.7)	28.6 (40.5)	23.9 (36.3)	30.1 (43.9
r.m.s.d. in bond lengths (Å)	0.012	0.006	0.006	0.003	0.005
r.m.s.d. in bond angles (deg)	1.9	1.3	1.3	1.1	1.2
Average Β (Å^2^)					
protein atoms (chains A, B, and C)	46.1	36.2^b^	33.4^b^	81.7, 86.4, 102.0	26.4^b^
water molecules	44.96	56.3^c^	48.5^c^	61.5	46.7^c^
ligand atoms (chains A, B, and C)			50.8^b^	170.0, 162.8, 183.1	47.7^b^
PDB entry	8R48	8R4K	8R4G	8R49	8R4J

a Values in parentheses are for the outermost shell.

b Due to the number of reflections, local NCS restraints were used during refinement; therefore, the *B* factor value reported is the same for all chains.

c Due to the number of reflections, local NCS restraints were used during refinement; therefore, the *B* factor value reported is the same for all water molecules.

## Results and Discussion

### Enzyme Characterization

Although we purified *st*Pho1, we noticed that over time, the enzyme sample showed proteolysis, which resulted in two segments of approximately equal molecular weight (∼50 kDa), as was evident from SDS PAGE analysis (Figure 1). We also investigated whether this proteolysis occurred in the crystallization experiments, and after thorough washing with the crystallization buffer, a single crystal was loaded in SDS PAGE. Analysis showed the same two bands we observed in the solution. When the structure of *st*Pho1 was determined (see below), a 65 amino acid fragment of the L78 region was not detectable within the electron density map. Since the molecular weights of the two segments produced after the removal of the L78 region were approximately 50 kDa, we concluded that the enzyme occurs in two forms: one where the polypeptide is intact with a molecular weight of 104 kDa and the other composed of two different polypeptide chains produced after the removal of a large portion of the L78 region. We termed the second form *st*Pho1ΔL78 and proceeded to characterize the enzymatic activity of both forms.

**Figure 2:**
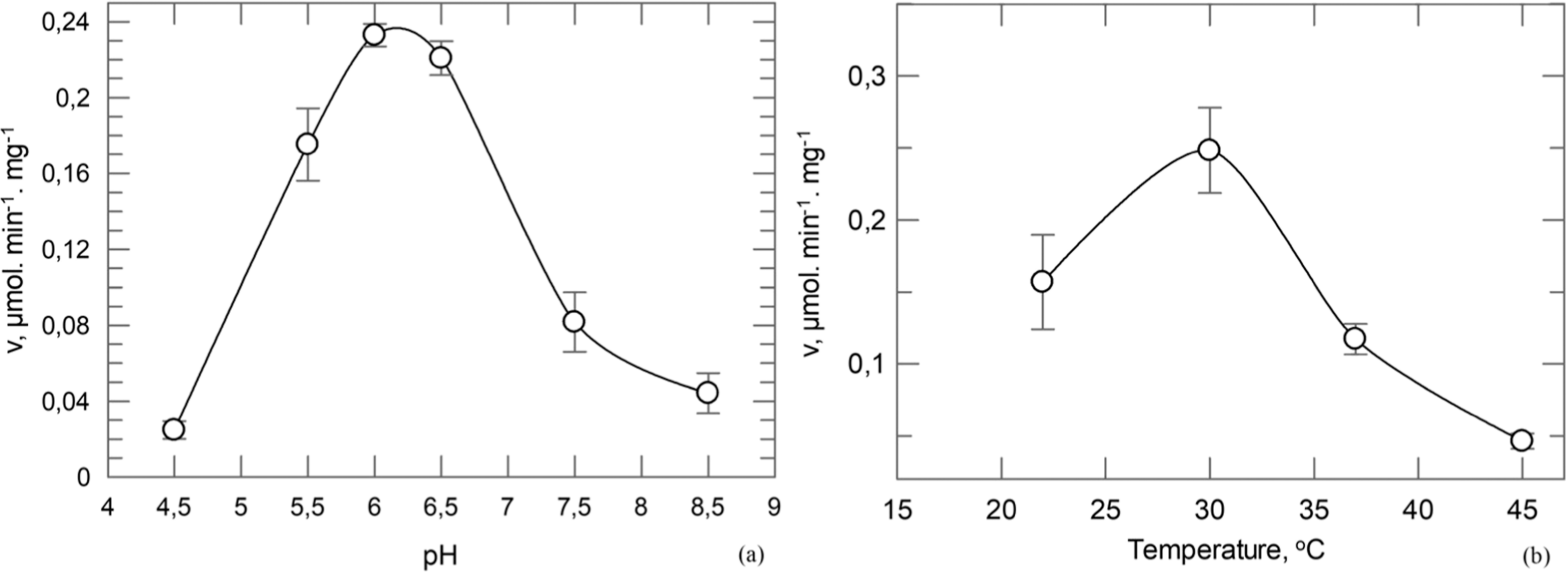
pH (a) and temperature (b) curves for stPho1 activity, together with standard error bars. Points represent the *k* value measured as described in the Materials and Methods section.

To establish the optimum pH and temperature for enzyme activity, we first measured the enzymatic activity in the 4.5–8.5 pH range. Kinetic studies were performed in the direction of glycogen synthesis using 50 μg/mL of *st*Pho1 using six different buffers (20 mM sodium acetate buffer for pH 4.5 and 5.5, 20 mM sodium citrate buffer for pH 6.0 and 6.5, and 20 mM Tris–HCl for pH 7.5 and 8.5) containing 60 mM KCl, 0.6 mM EDTA, and 0.6 mM dithiothreitol, using constant concentrations of glycogen (0.2% w/v) and Glc-1-P (14 mM) at 30 °C. As it is evident from Figure 2a, the enzyme exhibited optimum activity at pH 6.0 in the direction of glycogen synthesis, a value comparable to the values of 5.5 and 6.5 observed previously.^5,59^ To determine the optimum temperature for the activity, enzymatic assays were performed at temperatures 20, 30, 37, and 45 °C. Kinetic studies were performed in the direction of glycogen synthesis using 50 μg/mL of *st*Pho1 in a 20 mM sodium citrate buffer (pH 6.0) containing 60 mM KCl, 0.6 mM EDTA, and 0.6 mM dithiothreitol, using constant concentrations of glycogen (0.2% w/v) and Glc-1-P (4 mM). The optimum temperature was found to be 30 °C (Figure 2b), as previously reported.^5^ Both enzyme forms, *st*Pho1 and *st*Pho1ΔL78, follow the Michaelis–Menten model, and their kinetic constants are presented in Table 2, together with the respective values for rabbit muscle glycogen phosphorylase b (rmGPb) in the presence of 1 mM AMP. The *V*_max_ values of *st*Pho1 and *st*Pho1ΔL78 are similar, indicating that both forms are equally active. However, their *K*_m_ values for substrates Glc-1-P and glycogen differ significantly with the values for *st*Pho1ΔL78, almost half of those of *st*Pho1, indicating that abscission of the L78 segment increases the enzyme’s affinity for substrates. Interestingly, the values of the *K*_m_ for glycogen and the *K*_i_ for β-cyclodextrin of *st*Pho1ΔL78 are approximately half of that of *st*Pho1 (Table 1), indicating that L78 probably interferes with glucan binding to the enzyme. The inhibitory potency of glucose is similar for both forms of the enzyme, indicating that the active site of the enzyme is not affected by L78. Caffeine, an inhibitor binding at the inhibitor binding site at the animal enzymes,^60^ displays stronger potency (*K*_i_ is two times lower) for *st*Pho1ΔL78 than for *st*Pho1. This indicates the existence of an inhibitor binding site in *st*Pho1, like that in GP, and that L78 interferes with binding at this site. Comparing the *st*Pho1 kinetic constant values to those of rmGPb, it is evident that the *V*_max_ value is significantly lower, indicating that the *st*Pho1 enzyme has a lower reaction rate than the animal homologue, which is somehow expected since its natural substrate is starch and not glycogen. This is also evident when comparing the *K*_m_ values of *st*Pho1 and GP for glycogen (Table 2). Also, glucose and caffeine are much more potent inhibitors of the animal enzyme than for *st*Pho1. However, *st*Pho1 and *st*Pho1ΔL78 show a much stronger affinity for substrate Glc-1-P than for GP. A notable difference between GP and *st*Pho1 is the potency of β-cyclodextrin, which binds at the glycogen storage binding site of GP.^5^ The *K*_i_ value for GP is 192 and 451 times higher than those for *st*Pho1 and *st*Pho1ΔL78, respectively. This indicates that the plant enzyme has a much stronger affinity for β-cyclodextrin than the animal enzyme, which suggests significant differences in the polysaccharide binding site. Since mammalian muscle phosphorylase activity is dependent on the presence of the allosteric activator AMP,^35^ we investigated whether the presence of AMP affects the *st*Pho1 enzymatic activity. The effect was tested with 1 mM AMP, using 30 μg/mL of *st*Pho1 in 20 mM sodium citrate buffer (pH 6.0) containing 60 mM KCl, 0.6 mM EDTA, and 0.6 mM dithiothreitol, using a saturating concentration of glycogen (0.2% w/v) and various concentrations of Glc-1-P (3, 6, 10, and 15 mM). The *V*_max_ and *K*_m_ values were very similar to the values in the absence of AMP (Table 2), and therefore, it seems that the activity of *st*Pho1 is not affected by the presence of AMP like the human liver (hlGP)^34^ or brain GP (hbGP),^36^ in agreement with previous studies.^59^

**Table 2:** Kinetic Constants of *st*Pho1, *st*Pho1ΔL78, and rmGPb

	*st*Pho1	*st*Pho1ΔL78	rmGPb
*V*_max_ Glc-1-P (μmol/min/mg)	0.37 ± 0.01	0.32 ± 0.02	2.9^63^
*V*_max_ Glc-1-P (μmol/min/mg) + 1 mM AMP	0.39 ± 0.01		64^63^
*K*_m_ Glc-1-P (μM)	2.3 ± 0.2	1.4 ± 0.2	28^63^
*K*_m_ Glc-1-P (μM) + 1 mM AMP	2.4 ± 0.2		9.0^63^
*K*_m_ × 1000 glycogen (%)	114.2 ± 7.6	60 ± 9	12^1^^63^
*K*_i_ α-d-glucose (mM)	42.8 ± 2.6	38.4 ± 1.5	3.2^a^^63^
*K*_i_ caffeine (mM)	2.8 ± 0.2	1.4 ± 0.1	0.13^a^^63^
*K*_i_ β-cyclodextrin (μM)	73.1 ± 5.9	31.0 ± 1.0	14,100^a^^61^

a Values were measured in the presence of 1 mM AMP.

The inhibition constant values for α-d-glucose, caffeine, and β-cyclodextrin, three common GP inhibitors that bind at the catalytic,^62,63^ inhibitor,^64^ and glycogen storage binding sites^61^ of the mammalian enzyme, respectively, were measured (Table 2). It seems that α-d-glucose and caffeine are moderate inhibitors of both enzyme forms, while β-cyclodextrin is an order of magnitude more potent. Nevertheless, again the *st*Pho1ΔL78 values are significantly lower than those of *st*Pho1, indicating that abscission of the L78 segment provides better access for binding of these inhibitors.

### Structure of *st*Pho1ΔL78

**Figure 3:**
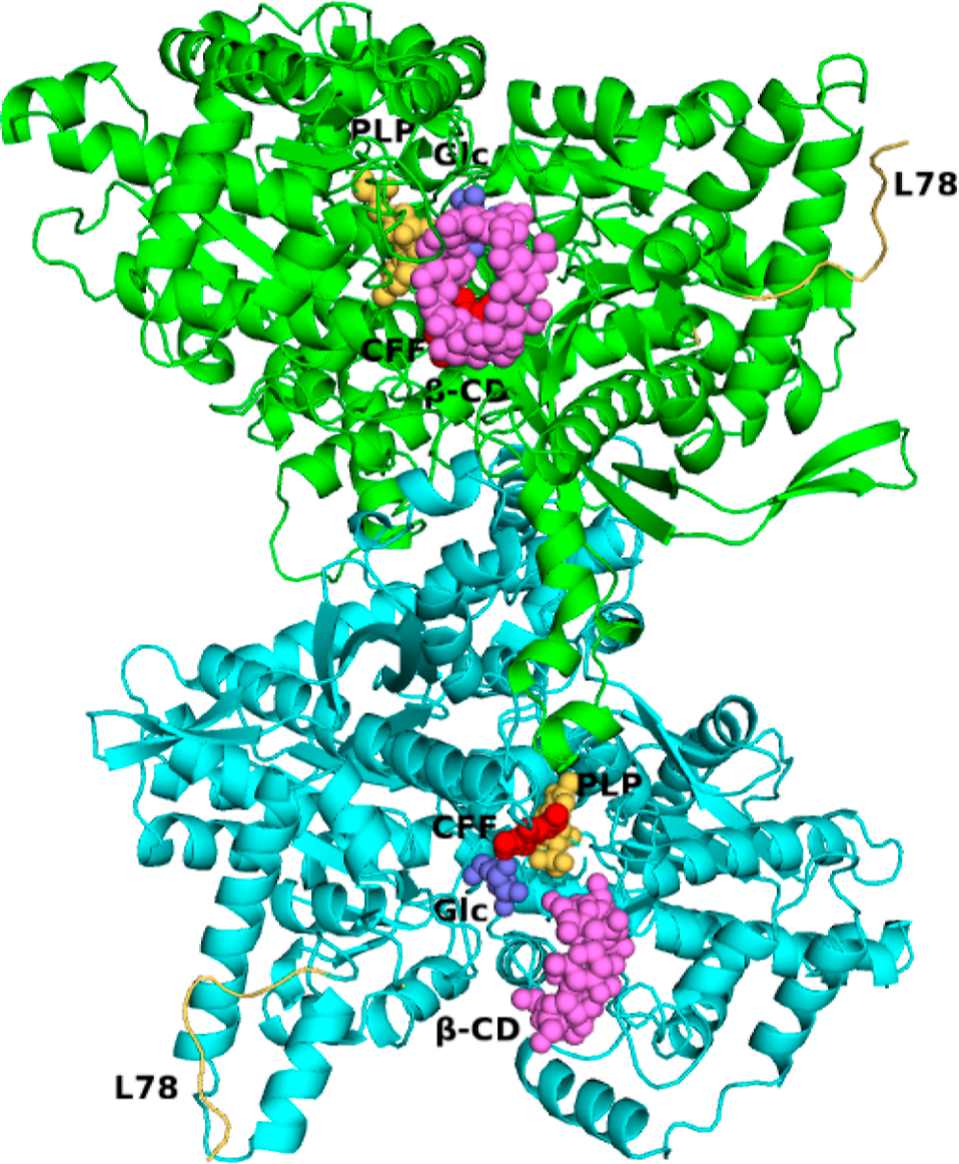
*st*Pho1ΔL78 dimer. Two subunits are shown in green and cyan, and the L78 segment is in gold. The active site, the inhibitor site, and the polysaccharide binding site are indicated by CPK models of PLP (yellow) and α-d-glucose (magenta), caffeine (red), and β-CD (purple), respectively.

**Figure 4:**
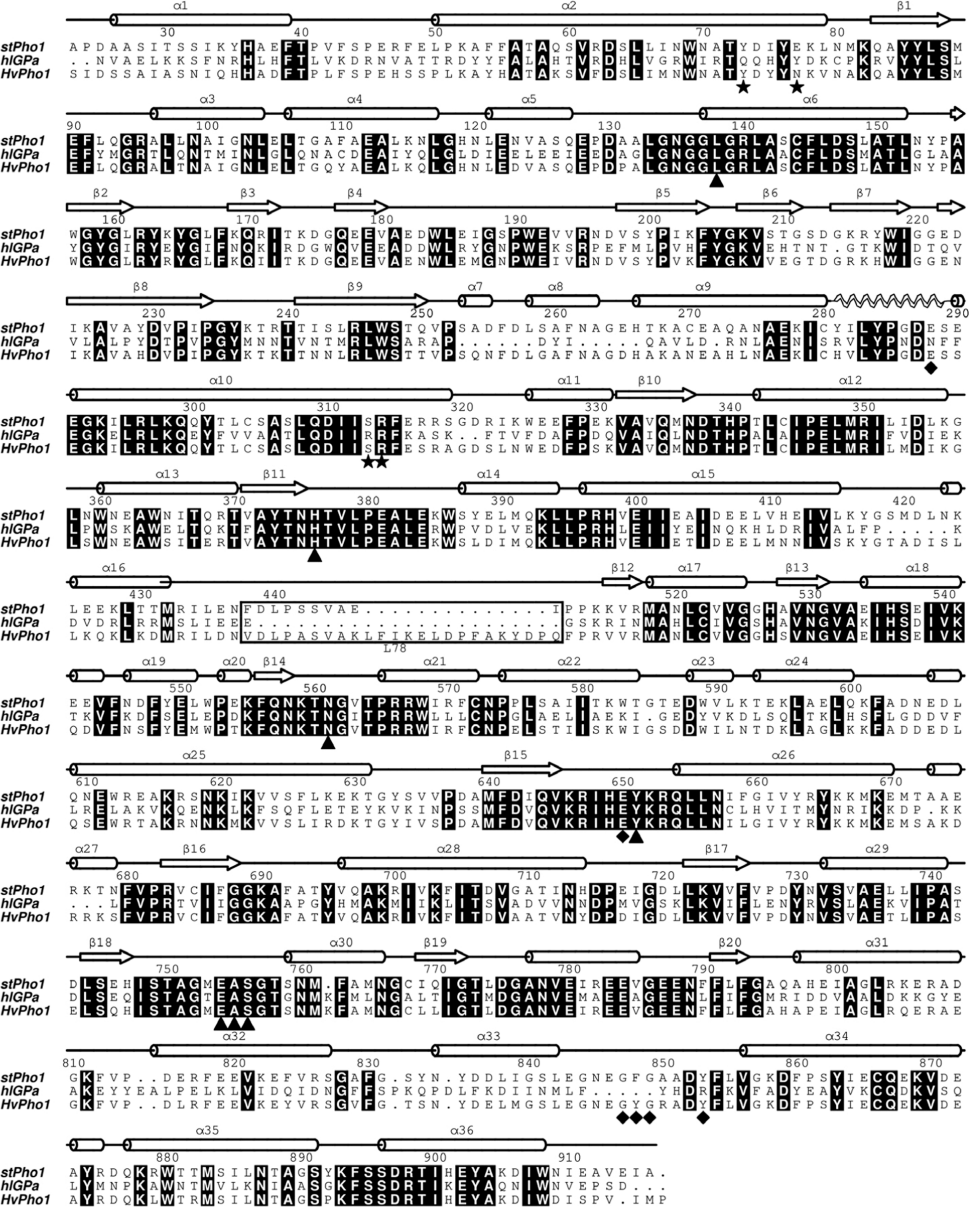
Structure-based sequence alignment of *hl*GPa (PDB entry 2ATI(66)) and the *Hv*Pho1 (PDB entry 5LR8(8)). Numbering follows the *st*Pho1 residue numbering; identical residues are shown in black boxes. Black triangles, stars, and diamonds indicate residues of the active, the allosteric, and the glycan binding sites, respectively, while the L78 is boxed. The secondary structure (α-helices as cylinders and β-strands as arrows) of stPho1 is also shown above the sequence (the 280s loop is drawn as a spiral).

The free structure was solved from two different crystal forms that belonged in space groups *P*3_1_21 and *C*2 (Table 2). In *P*3_1_21, there is one *st*Pho1ΔL78 molecule in the asymmetric unit, whereas in *C*2, there are three. *st*Pho1ΔL78 structures from the two different crystal forms are almost identical, and since the structure from the crystal that belonged to *P*3_1_21 was resolved at higher resolution (2.2 Å), we focused our analysis to this structure. stPho1 is composed by 916 residues.^17^ Except for 23 residues at the N-terminus and 66 residues of the L78 region, all other residues are well located within the electron density map. PISA,^65^ size-exclusion chromatography, and native gel electrophoresis indicated that the biological assembly is a homodimer (Figure 3). The second molecule can be generated by applying the rotation matrix −*x*, *y* – *x*, and −*z* and the transformation matrix 2, 1, and −2/3 to the fractional coordinates. The dimer interface surface is 60,640 Å^2^ and buries an area of 5980 Å^2^. The two subunits are held together by 26 hydrogen bonds, 6 salt bridges, and numerous van der Waals interactions. The monomer is composed of two separate proteolytically produced segments, the N-terminal (residues 23–446) and the C-terminal (residues 511–916). The two segments are held together by 27 intersegment hydrogen bonds and 288 van der Waals interactions. The catalytic site is formed at the domain interface. The essential cofactor, pyridoxal phosphate (PLP), is attached by a covalent Schiff base linkage to Lys762 in the C-terminal domain (Figure 3). Each segment is centered on an α/β structure, while both N- and C-terminal segments contain 15 and 21 α-helices, respectively (Figure 4). The first 22 residues of the polypeptide chain were not located within the electron density map, and the structure begins with the α1 helix (residues 25–39) followed by a loop connecting helices α1 and α2. This loop is termed the cap region in the human GP structures, and it is part of the interface upon dimer association.^35^ The α1 helix packs against the start of α2, with the remainder of the α helix packing against strands β4, β7, β10, and β11 of the central β-sheet core. Leading from α2, the polypeptide chain forms the central β-strand, β1. Following β1, the chain enters a bundle of three helices, α3, α4, and α5. The α4 packs against α1, while α5 is associated with the N-terminus of α2. The helices α1 and α4 and the N-termini of both α2 and α5 form a compact independent subdomain, termed the cap/α2 subdomain. The α5 helix leads to α6 and then to β2, which packs closely against the β-sheet core. The chain then enters an antiparallel β-sheet formed by β3 and β4, leading into the peripheral strand of the core, β5. The β5 strand is antiparallel to β8 and joined to it via an antiparallel β-sheet formed from β6 and β7. This sheet produced the second major excursion from the core. The β8 strand, antiparallel to β9, is linked to a helical bundle formed from α6, α7, and α8, termed the tower subdomain. This subdomain forms the most significant excursion from the core, anchored at its base by a long helix (α9, residues 291–319), which is situated at the core of the N-terminal segment. The loop connecting a8 to α9 is the structural equivalent of the mammalian GPs 280s, which forms a gate to the catalytic site. Residues of helix α9 are the structural equivalents of the mammalian GPs that produce the AMP allosteric site located at the dimer interface. The chain then forms a loop that develops to a short helix, α10, and subsequently joins the β-sheet core at β10. At the end of β10, the chain leads to α11 and α12 before joining the sheet at β11. The loop connecting β11 to α13 forms part of the catalytic site. The helix α13 then leads to helices α14 and α15, packed against each other, while the end of α15 leads to a loop that is part of the L78 region. The polypeptide chain enters the C-terminal segment from the L78 loop with a sheet–helix–sheet motif consisting of strands β12, β13, and β14 and helices α16, α17, α18, and α19. This leads to a compact bundle of five helices, consisting of α21–α25. The C-terminus of α25 leads to a core of six parallel β-strands, β15–β20, at the center of the C-terminal segment. Flanked on one face are three helices, α26, α27, and α28, and on the opposite face of the sheet are α29, α30, and α31. The chain then leads to a β-strand (β20) and then folds into an α-helical region, in which α-helices α32–α34 and the N-terminus of α35 form one unit and helices α35–α37 form a second unit.

The *st*Pho1ΔL78 structure is similar to the hlGPa (PDB entry 2ATI(66)) and the *Hv*Pho1 (PDB entry 5LR8(8)) structures with r.m.s.d. values of 6.5 and 0.7 Å for 795 and 807 equivalent Cα positions, respectively.

**Figure 5:**
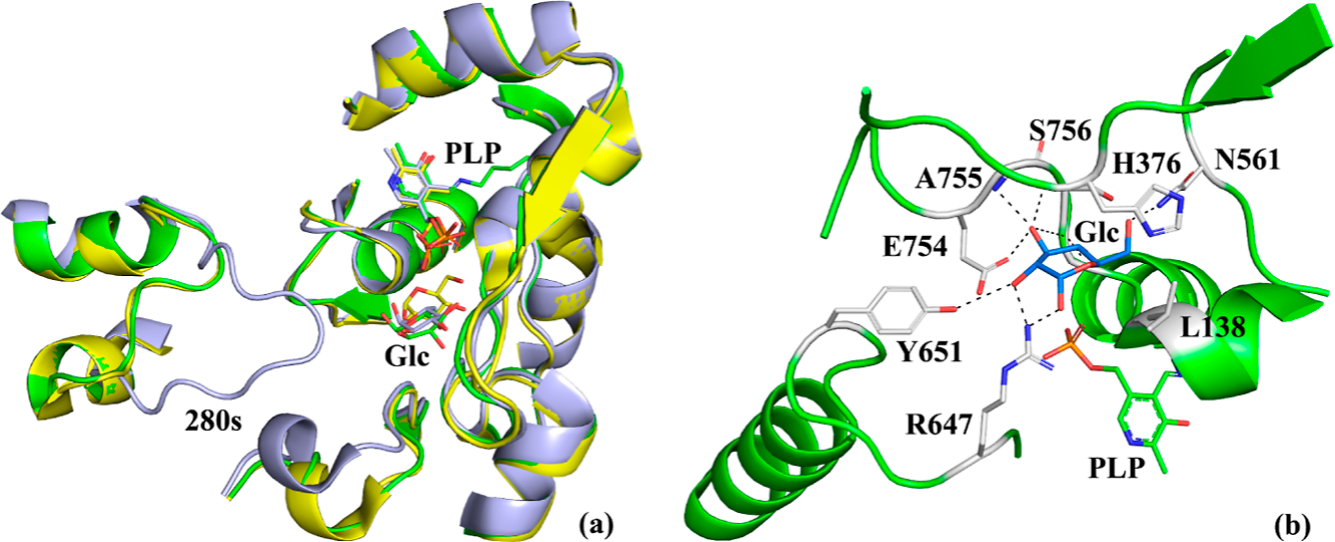
(a) Superposition of the *st*Pho1ΔL78 structure (green) onto the hlGPa-AMP (PDB entry 1FA9;^67^ yellow) and hlGPa-glucose (PDB entry 2ATI;^66^ gray) structures. PLP, α-d-glucose (Glc), and the 280s loop are labeled. (b) Binding of glucose to the *st*Pho1ΔL78 structure. Hydrogen bonds are shown as dashed lines.

The most significant differences lie within the 280s loop (residues 280–289 of hlGPa, a flexible loop that controls entrance to a long channel leading to the active site^31^), and the L78 segment. The conformation of the 280s loop in the *st*Pho1ΔL78 structure is similar to that in the active form of human liver GPa (hlGPa) in complex with AMP and α-d-glucose (PDB entry 1FA9(67)) but very different to that of the hlGPb in complex with α-d-glucose (PDB entry 2ATI(66)) (Figure 5). Therefore, it seems that the 280s loop in *st*Pho1ΔL78 adopts a conformation that agrees with an active form of the enzyme.

There seems to exist a controversy with respect to the L78 insertion peptide. Based on the initial alignment of the *st*Pho1, rmGPb, and *E. coli* GP sequences,^17^ the L78 peptide was considered to be residues 414–491. In *Hv*Pho1, the L78 peptide is considered as residues 483–561.^8^ However, superimposition of the structures of *st*Pho1 and *Hv*Pho1 (PDB entry 5LR8(8)) onto the rmGPb structure (PDB entry 3E3L(35)) revealed that the L78 peptide actually spans residues 438–511 for *st*Pho1, 4 residues shorter than 78, and 483–563 for *Hv*Pho1, 3 residues longer than 78 (Figure 4). The position of 9 residues 438–446 at the N-terminus residue 511 at the C-terminus of the L78 peptide was determined in the *st*Pho1ΔL78 structure. The N-terminal residues form a loop stemming from a helix (residues 423–432), which protrudes toward the solvent (Figure 2). The conformation of a portion of the L78 (13 residues at the N-terminus and 10 residues at the C-terminus) has been also observed in the *Hv*Pho1 structure (PDB entry 5LR8(8)) and was found to form solvent-oriented loops.

The catalytic site is a deep cavity located at the center of the molecule, 15 Å from the protein surface and close to the essential cofactor PLP (Figure 2). The architecture of this site has been probed with α-d-glucose and glycopyranose-derived inhibitors.^60^ Comparing the active sites of rmGPb (PDB entry 3E3L(35)), hlGPa (PDB entry 1FA9(67)), and *st*Pho1ΔL78, it seems that the active site is structurally conserved. The only significant difference is the 280s loop, which adopts a totally different conformation in *st*Pho1ΔL78 from that in the rmGPb-glucose (PDB entry 2GPB(62)) or the hlGPa-glucose (PDB entry 2ATI(66)) complexes but very similar to the activated hlGPa structure (PDB entry 1FA9(67)) (Figure 5a). The binding of inhibitors at the active site of GP, which inactivates the enzyme, stabilizes the inactive T-state conformation of the enzyme and induces a conformational change of the 280s loop from an open to a closed conformation toward the catalytic site.^60^ This conformational change signals the deactivation of GP.^31,67^ However, this does not happen at *st*Pho1ΔL78 upon binding of α-d-glucose, and the structurally equivalent 280s loop maintains the same open conformation as in the free enzyme (Figure 5b). This open conformation was also observed at the *Hv*Pho1 (PDB entry 5LR8(8)).

**Figure 6:**
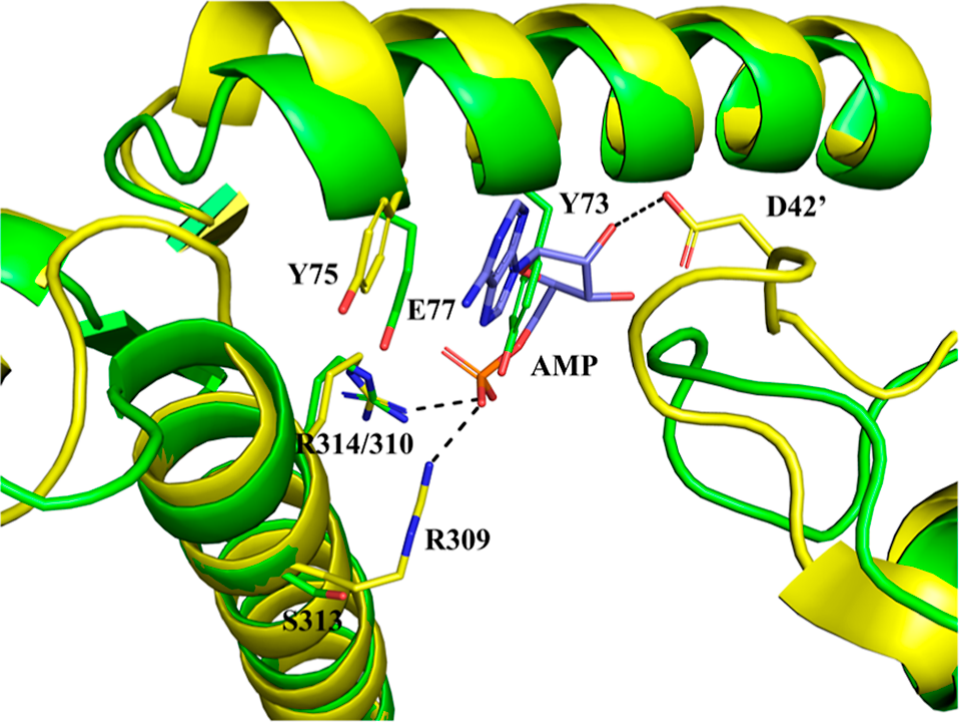
Superposition of the *st*Pho1ΔL78 structure (green) onto the hlGPa-AMP complex structure (PDB entry 1FA9;^67^ yellow). The hlGPa residues that form interactions with the nucleotide together with their *st*Pho1 structural equivalents are shown as sticks and they are labeled. Residues from the symmetry-related subunit are labeled with a prime.

Mammalian GPs are allosteric enzymes^31^ following the Monod–Wyman–Changeux model for allosteric proteins.^37^ Allosteric activators or inhibitors bind to a site termed the allosteric site,^31,67^ formed at the subunit interface of the dimer, by a loop from one subunit termed the cap residues (residues 42′-45′ of hlGPa) and a helix from the other (residues 48–78). In *st*Pho1, the allosteric activator, AMP, does not have any effect on its activity (Table 2). The allosteric site of GP recognizes a variety of phosphorylated compounds such as IMP (weak activator), ATP, glucose-6-P, NADH, UDP-glucose, 2-deoxy-glucose-6-P, β-glycerophosphate, and inorganic phosphate.^31^ The allosteric site is not conserved in *st*Pho1ΔL78 in sequence (Figure 4) and in structural terms. Superposition of the hlGPa-AMP complex structure (PDB entry 1FA9)^67^ onto the *st*Pho1ΔL78 structure (Figure 6) reveals significant structural differences, with the most striking being that of Tyr73 whose side chain passes through the ribose of AMP. Therefore, it appears that AMP or any other nucleotide cannot bind at this site in *st*Pho1, which may explain the inability of AMP to affect the *st*Pho1 enzymatic activity.

**Figure 7:**
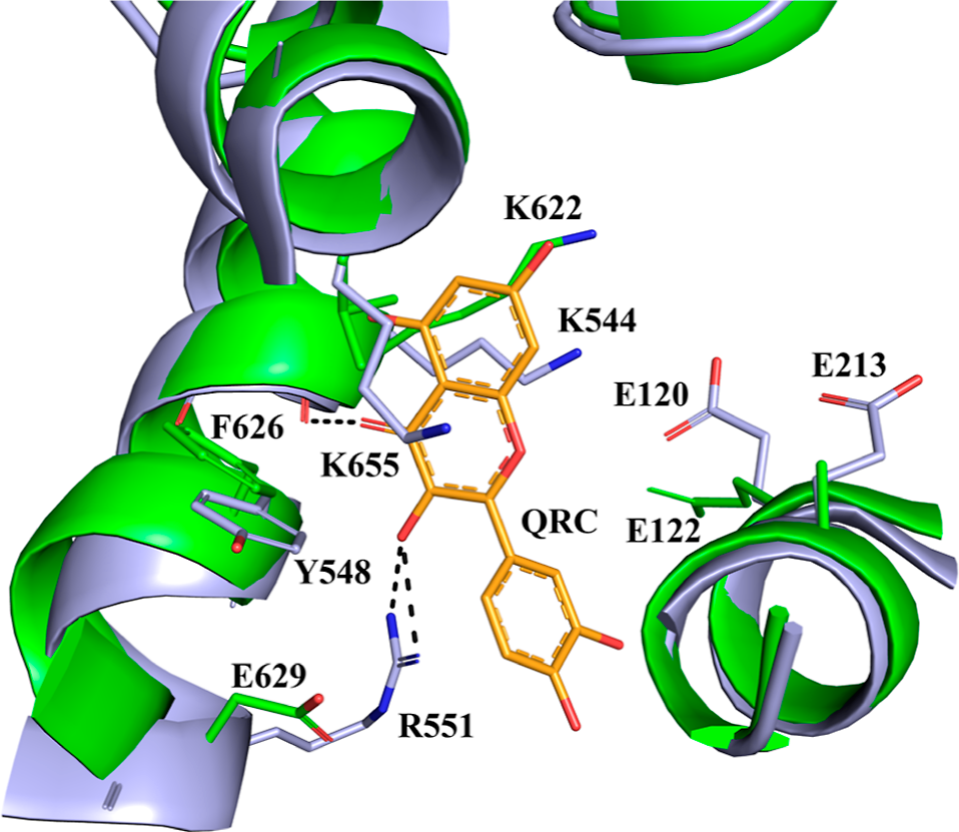
Superposition of the ΔstPho1 structure (green) onto the rmGPb-quercetin complex (PDB entry 4MRA;^69^ magenta) structure in the vicinity of the quercetin (QRC) binding site. Residues interacting with quercetin (yellow) together with their structural equivalents in ΔstPho1 are shown as sticks and labeled. Hydrogen bonds are presented as dashed lines.

Human liver GP is a validated pharmaceutical target for the discovery of novel antihyperglycemic drugs.^68^ Two of the most promising sites for drug discovery are the quercetin binding site^69^ and the indole binding site.^70^ The quercetin binding site in GPs^69^ is located 15 Å away from the active site, 43 Å from the allosteric site, and 32 Å from the inhibitor site. It is composed of a shallow groove formed by Lys544, Arg551, Lys655, and Tyr548 at one side and Glu120 and Glu123 at the other side (Figure 7). The quercetin binding site is the site with the least conservation between the muscle, the liver, and the brain human isozymes.^69^ Quercetin is a potent inhibitor of the mammalian enzyme with *K*_i_ values 32–69 μM.^71^ However, quercetin did not have any effect on the enzymatic activity of *st*Pho1 up to a concentration of 1 mM. The equivalent to the quercetin binding site in *st*Pho1 is partially conserved (Figure 7). Thus, out of the six residues that constitute this site, only Lys544 and Glu120 of rmGPb are conserved in *st*Pho1 as Lys622 and Glu122. The rest of the residues, Glu123, Lys655 and Tyr548, of rmGPb are Ala124, Leu737, and Phe626 in *st*Pho1. Arg551 of rmGPb does not have a structurally equivalent residue in *st*Pho1 (Figure 7). These differences may offer a structural explanation for the inability of quercetin to affect the *st*Pho1 activity.

**Figure 8:**
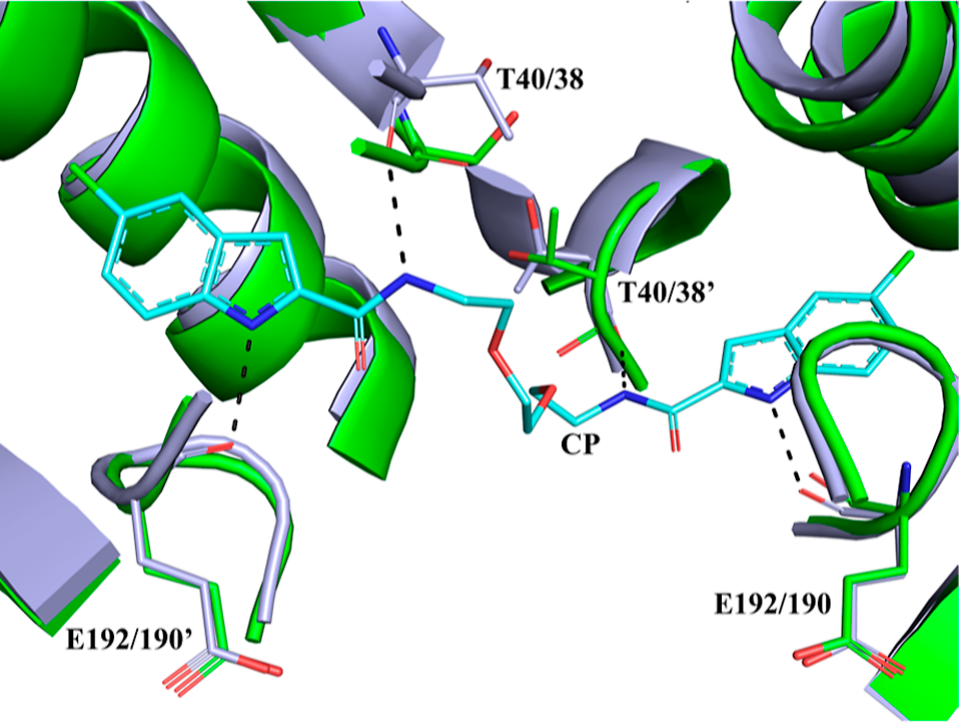
Superposition of the *st*Pho1ΔL78 (green) structure onto the hlGPa-CP-526,423 (PDB entry 1EM6;^70^ gray) structure in the vicinity of the indole binding site. The inhibitor molecule CP (cyan) and the interacting residues together with their structural equivalents in ΔstPho1 are shown as sticks, while hydrogen bonds are presented as dashed lines. Residues from the symmetry subunit are labeled with a prime.

The new allosteric or indole binding site in mammalian GPs is located inside the central cavity formed by the association of the two GP subunits, 15 Å from the allosteric site, 33 Å from the catalytic site, and 37 Å from the inhibitor site.^31^ At this site, some of the most potent GP inhibitors (*K*_i_s at the lower nM range), which also had a significant effect in lowering blood glucose levels in animal models, were found to bind.^70,72^ This site is conserved in both sequence and structural terms in *st*Pho1ΔL78 (Figure 8).

Apart from the binding sites described above, three other sites with special interest exist in GPs: the catalytic, the inhibitor, and the glycogen storage site. X-ray crystallography of three different *st*Pho1ΔL78-ligand (glucose, caffeine, and β-CD) complexes revealed the existence of these sites to *st*Pho1. The structures of these complexes have been resolved at medium to low resolution, 3.1, 3.7, and 2.8 Å, for glucose, caffeine, and β-CD complexes, respectively. To avoid any bias in the modeling of the ligands in the electron density maps, the presence of each ligand within the electron density maps has been verified by polder omit maps.^73^ Although the resolution attained for these complex structures does not permit the inference of detailed structural information on the binding mode, they have provided substantial insights for each binding site such as its location and architecture. Below, we analyze the binding of each of those ligands and their interaction pattern with *st*Pho1ΔL78.

### Binding of α-d-Glucose

The binding of α-d-glucose is very similar to its binding to hlGPa (PDB entry 2ATI),^66^ rmGPa (PDB entry 1LWN),^74^ rmGPb (PDB entry 2GPB),^62^ and hmGPa (PDB entry 1Z8D).^75^ The inhibitor binds close to PLP (∼4 Å), and its binding does not cause any significant conformational change, with one exception. Thus, the hydroxyl groups of glucose are in hydrogen-bonding distance from residues Asn561, Tyr651, Arg647, Glu754, Ala755, Ser756, and Gly757 (Figure 5b). Glucose also could engage in 23, 22, and 29 van der Waals interactions with *st*PhoΔL78 residues in molecules A–C of the asymmetric unit, respectively. In comparison, α-d-glucose upon binding to rmGPb (PDB entry 2GPB)^62^ forms nine hydrogen bonds with residues Asn284, His377, Asn484, Glu672, Ser674, and Gly675, and 26, 24, and 30 van der Waals interactions in hlGPa (PDB entry 2ATI),^66^ rmGPa (PDB entry 1LWN),^74^ and hmGPa (PDB entry 1Z8D),^75^ respectively. As mentioned above, the active site of *st*Pho1ΔL78 is partially conserved with GPs. Two significant differences involve Asn284 and His377. The binding of glucose to mammalian GPs induces a conformational change of the 280s loop, which closes the active site forming a pocket.^66^ This conformational change has not been observed in the *st*Pho1ΔL78-glucose complex, and the glucose interactions with Asn284 (from the 280s loop) are replaced by those with Arg647, whose side chain undergoes a significant conformational change by rotating ∼180° from its position in the free structure. One important residue for binding of all GP glucose-based inhibitors is His377, which always forms hydrogen bond interactions with some of the most potent glucose-based inhibitors.^31,60^ The structural equivalent of GP’s His377 in *st*Pho1ΔL78 is His376. The conformation of this residue is not conserved in *st*Pho1ΔL78, where it adopts a conformation away from the ligand and therefore cannot form any interactions. The partial conservation of the *st*Pho1ΔL78 active site is also evident when comparing the solvent accessible protein surface area, which becomes inaccessible upon ligand binding to hlGPa^66^ and to *st*Pho1ΔL78. This value is 128 Å^2^ for the hlGPa-glucose complex, whereas for the *st*Pho1ΔL78 glucose complex, it is 367 Å^2^. These values correlate well with the surface of glucose, which becomes buried upon binding to the two proteins 90% (*st*Pho1ΔL78) and 96% (hlGPa), indicating that glucose is more exposed to the solvent when bound to *st*Pho1ΔL78 than when bound to hlGPa (PDB entry 2ATI).^66^ The energy cost for the conformational change of Arg647, the nonconservation of His376 conformation, and the solvent exposure of glucose in *st*Pho1ΔL78 may offer a structural explanation for the 10 times difference between the *K*_i_ values of glucose for *st*Pho1 and rmGPb (Table 2).

### Binding of Caffeine

**Figure 9:**
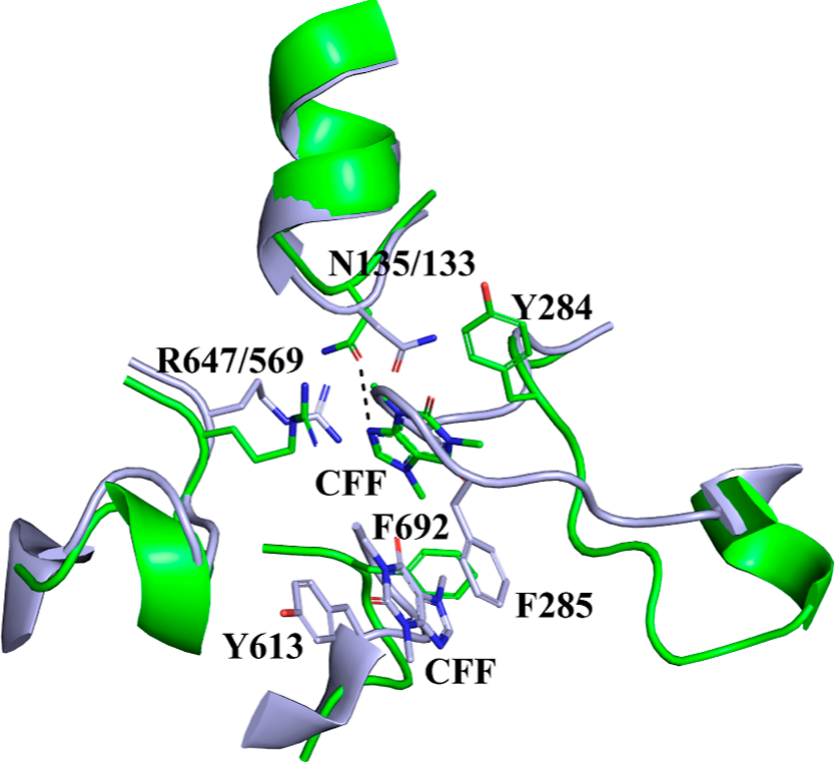
Binding of caffeine to Δ*st*Pho1 (green). The structure of the hlGPb-caffeine complex (PDB entry 1L7X;^77^ gray) is also shown superimposed. Residues interacting with the caffeine molecule (CFF) are shown as sticks, and the hydrogen bonds between caffeine and Asn135 are presented as dashed lines.

In mammalian GPs, the inhibitor site is conserved and constitutes a hydrophobic binding pocket, on the surface of the enzyme, 12 Å from the catalytic site.^31^ X-ray crystallography in rmGPb and rmGPa has revealed binding at this site of purines (e.g., adenine and caffeine), nucleosides, nucleotides, NADH, FMN, FAD, riboflavin,^31^ and several flavonoids.^76^ Caffeine has been also shown to bind in hlGPb and hlGPa, while the binding of riboflavin and uric acid has been observed in hlGPa.^77^ The inhibitor site, in the T state mammalian enzymes, is formed by two hydrophobic residues, Phe285, from the 280s loop, and Tyr613, from a helix (residues 613–631). The phenyl rings of these two residues stack to each other, forming the inhibitor site. Occupation of this site stabilizes the T-state conformation of the enzyme and blocks access to the catalytic site, thereby inhibiting the enzyme. Glucose can bind simultaneously, at the catalytic site, and inhibit enzyme activity synergistically with caffeine.^78^ In *st*Pho1, Tyr613 is conserved as Tyr695, but there is no structurally equivalent residue for Phe285 (Figure 4). Furthermore, as mentioned before, the conformation of the 280s loop is significantly different between the two enzymes. As a result, the hydrophobic pocket of the inhibitor site observed in GPs is no longer formed. Caffeine inhibits *st*Pho1 and *st*Pho1ΔL78 (Table 2) but displays a 21- to 22-fold selectivity in favor of rmGPb. The *st*Pho1ΔL78-caffeine complex was produced by soaking *st*Pho1ΔL78 crystals in a caffeine (7.5 mM) solution. The caffeine molecule was found bound to only one of the three protein molecules of the asymmetric unit. Since the resolution of X-ray diffraction data was limited to 3.7 Å, omit and polder maps were used to validate whether the density corresponded to a caffeine molecule. The correlation coefficient CC(1,3) was larger than CC(1,2) and CC(2,3), indicating that the density most likely corresponded to the atomic features of the polder omit selection (caffeine).^73^ The binding location of caffeine in *st*Pho1ΔL78 is significantly different from that observed in mammalian enzymes. Caffeine binds ∼8 Å away from the position it occupies when bound to hlGPb (PDB entry 1L7X(77)) (Figure 9). The caffeine molecule packs against the phenyl side chain of Tyr284 and is almost perpendicular to the phenyl ring of Phe692. Although at 3.7 Å resolution, the modeling of residue side chains has a certain degree of uncertainty, caffeine upon binding to *st*Pho1ΔL78 is in hydrogen bonding distance from the side chain of Asn135 (N9-OD1 distance 3.3 Å) and may form 7 van der Waals interactions with Arg647 and Phe692 (Figure 8). This binding mode appears to be rather weak compared to that in hlGPb (PDB entry 1L7X),^77^ hlGPa (PDB entry 1L5Q),^77^ and rmGPb (PDB entry 1GFZ),^64^ where it forms 70, 62, and 61 van der Waals contacts, respectively. The significant difference in the number of van der Waals contacts of caffeine in *st*Pho1ΔL78 and mammalian GPs complex structures may explain the inhibitory potency differences between *st*Pho1 and these enzymes (Table 2).

### Glucan Binding Site

**Figure 10:**
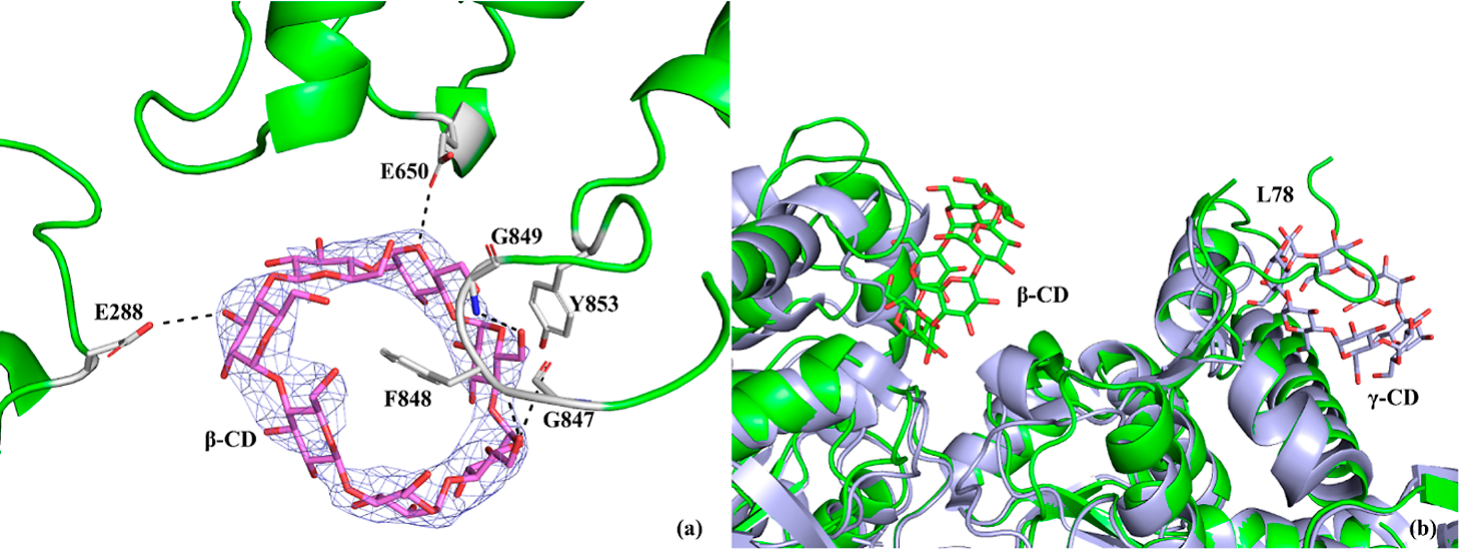
(a) Binding of β-CD to *st*Pho1ΔL78 in the crystal. The electron density of the β-CD molecule is also shown. (b) Structural superposition of the *st*Pho1ΔL78-β-CD complex (green) onto the rmGPb-γ-CD complex (PDB entry 1P2G;^61^ gray). Cyclodextrin molecules and interacting protein residues are shown as sticks, while hydrogen bonds are presented as dashed lines.

The glycogen binding site of rmGPb is situated on the surface of the protein molecule, approximately 30 Å from the catalytic site, and it is the region where glycogen is attached to GP in vivo.^60^ This site is composed of two helices (residues 396–418 and 420–429) and a loop connecting two antiparallel strands (residues 430–432 and residues 437–411). The binding of α-, β-, and γ- cyclodextrins (CD) with *K*_i_ values of 47.1, 14.1, and 7.4 mM for rmGPb, respectively, acarbose (*K*_i_ = 26 mM for rmGPa), maltopentaose (G5), and maltoheptaose (G7) (*K*_i_ = 1 mM for rmGPb) has been studied by kinetics and X-ray crystallography.^61,79,80^ The binding of cyclic and linear ligands at this site is supported by hydrogen bond interactions to Ser429, Lys473, and Asn407 and water-mediated interactions with Gln401, Arg426, Val431, and Gln433.^61^ In *st*Pho1, β-CD binds almost 200–450 times stronger than in rmGPb (Table 2). In the crystal structure of the *st*Pho1ΔL78-β-CD complex, one β-CD molecule was found in each one of the three molecules of *st*Pho1ΔL78 (Figure 10a) crystallographic trimer with *B* factors around 170 Å^2^. Although this value may seem rather high, it is only two times higher than the Wilson *B* factor of the *st*Pho1ΔL78-β-CD complex X-ray diffraction data (Table 2), which is normal for protein-bound ligands.

The β-CD molecule binds at a totally different location from the glycogen storage binding site in rmGPb (Figure 10b). The β-CD binding site (named glucan binding site thereafter) is situated on the surface of the protein molecule approximately 20 Å from the catalytic site. It is a shallow cavity composed of three loops. The first loop (residues 843–855) connects two helices (residues 836–842 and 856–875), the second (residues 646–649) connects a helix (residues 650–669) to a β-strand (residues 640–645), and the third (residues 687–695) connects a β-strand (residues 683–688) to a helix (residues 696–714). The *st*Pho1 glucan binding site is at the entrance of a channel leading to the active site and β-CD is bound at the one edge of this channel encapsulating the side chain of Phe848 where the other part of β-CD packs against the side chain of Tyr853 (Figure 10a). There, the glucose residues of β-CD, G2, G3, and G4 are involved in five hydrogen bonds with the main chain atoms of Gly847, Phe848, and Gly849 and the side chain of Glu650. Furthermore, G2–G6 are involved in 55 van der Waals interactions with *st*Pho1ΔL78 residues. We cannot rule out the involvement of L78 in β-CD binding since the C-terminus of L78 is 15 Å away from β-CD and we do not know the conformation of 64 residues there. The binding of β-CD seems to obstruct the entrance to the channel that leads to the active site. Hence, the strong inhibition displayed by β-CD for stPho1 (Table 2) could be attributed to this. The glycogen storage site of rmGPb is conserved in structural terms in *st*Pho1ΔL78. However, structural superposition of the rmGPb-γ-CD structure (PDB entry 1P2G)^61^ onto the *st*Pho1ΔL78 structure (Figure 10b) reveals that the γ-CD molecule superimposes onto a loop (residues 437–446) of *st*Pho1ΔL78, which is part of the L78 peptide. This observation implies that the L78 segment impedes any binding of glucan to *st*Pho1 in the corresponding location of the glycogen storage site of mammalian GPs. Binding to the *st*Pho1 glucan binding site of acarbose and maltotetraose has been observed in *Hv*Pho1.^8^ There, the four saccharide units of acarbose and the first three glucose units of maltotetraose were found bound at the places where G3, G4, and G5 units of β-CD bind at *st*Pho1ΔL78. The fourth unit is bound inside the channel, leading to the active site (11 Å away from PLP). In the *Hv*Pho1 structure, acarbose goes through a gate formed by Tyr900 and Tyr905 like in *st*Pho1ΔL78, where the structurally equivalent residues are Phe848 and Tyr853, respectively. In the *Hv*Pho1 structure, the binding of maltotetraose triggers a conformational change of a loop between Thr422 and Ala427 from an open to a more closed conformation,^8^ but such a conformational change is not observed in the *st*Pho1ΔL78 structure upon binding of β-CD and the corresponding loop remains in the open conformation.

### Proteolytic Cleavage of the L78 Region

The two segments produced by the proteolytic cleavage of the L78 region can form an active enzyme, as shown by the kinetic analysis (Table 2). The crystal structure of stPho1ΔL78 indicates that the two fragments are complementary and that the enzyme native conformation is unaffected by the break. The removal of the L78 region produces an enzyme that is 1.5 times more active than the nonproteolyzed enzyme and displays stronger affinities for Glc-1-P, glycogen, α-d-glucose, caffeine, and β-CD (Table 2). This proteolytic degradation has been also shown in mature potato tubers,^38^ sweet potato roots,^81^ the wheat endosperm Pho1,^39^ the rice endosperm Pho1,^82^*Hv*Pho1,^8^ and bovine liver phosphorylase.^83^ In bovine liver phosphorylase, proteolysis has been observed to happen even in the crystal, and SDS PAGE analysis revealed the presence of both the intact enzyme and the two proteolytic fragments in the crystal.^83^ The enzyme from the redissolved crystals displayed activity, but since intact enzyme was also present, it is not clear whether the proteolytic fragments can form an active enzyme. This could have been shown if the structure of bovine GP was known, but no structural data for this enzyme have been reported thus far. Proteolytic degradation of rmGPb results in two fragments that did not display any activity.^84^ Thus, it seems that this proteolysis occurs only in plant enzymes.

On the sweet potato roots enzyme (*Ib*Pho1),^85^ the L78 segment is proteolytically removed by proteasome 20S. However, in contrast to *st*Pho1ΔL78, the proteolytically modified *Ib*Pho1 displays lower binding affinity toward Glc-1-P and polysaccharides. Furthermore, the degradation of *st*Pho1 we observed in vitro cannot be attributed to proteasome 20S since it did not show in our SDS page of the purified enzyme (Figure 1).

Various studies have also shown a complete removal of the L78 insert.^38,40,81^ In the rice endosperm, Pho1 enzyme removal of the L78 segment affected neither the enzyme’s catalytic rates nor its affinities toward various substrates and inhibitors, but it contributed to heat stability.^86^ The L78 peptide contains potential phosphorylation sites, polyproline, and PEST regions (serving as a signal for rapid degradation^87^), which are rich in proline, glutamic acid, serine, and threonine. Wheat Pho1 is phosphorylated and can form multiprotein complexes with the phosphorylated starch branching enzymes.^88^ However, we have not observed any phosphorylation in the L78 part, which is defined in the *st*Pho1ΔL78 structure. Thus, it seems that in *S. tuberosum*, the proteolytic degradation has a different role from that in sweet potatoes, rice, and wheat starch phosphorylases. The removal of L78 has been previously shown to activate *Hv*Pho1 since it has been reported that an active unit of the enzyme is formed when a recombinant enzyme that lacked the L78 peptide was used.^8^ Furthermore, like in *st*Pho1, *Hv*Pho1 also undergoes proteolysis in the crystallization drop.^8^ The basis for this degradation is not clear. It will be either enzymatic or spontaneous. Since our protein sample is purified from a natural source, we cannot rule out the existence of traces of a protease (not detectable in SDS page). However, recombinant *Hv*Pho1 degrades over time in two protein segments.^8^

The involvement of *st*Pho1 under physiological conditions in both the synthesis and degradation of glucans has been shown.^2,18^ It has been reported that *st*Pho1 is capable of synthesizing glucans in a primer-independent way (i.e., in the absence of any glucan primer) from Glc-1-P.^46,89^ This activity is abolished by an antibody specific to the L78 peptide,^90^ implying that L78 has a critical role in regulating the involvement of *st*Pho1 in a catalytic pathway. Depending on the ratio Glc-1-P/orthophosphate ions, *st*Pho1 can be involved in starch biosynthesis or degradation. This route could be regulated by the L78 region. If the L78 region is present, then *st*Pho1 is involved in the degradation of the glucans. This is supported by our structural data, which indicate a possible involvement of this region in the binding of β-CD and therefore to any glucan binding to the enzyme. The proteolytic removal of the L78 regions directs *st*Pho1 to synthesize starch rather than degrade it. Our findings support this suggestion since our kinetic experiments were performed in the direction of glycan synthesis, and the *st*Pho1ΔL78 form of the enzyme displayed 1.5 times higher activity and 2 times greater affinity for glycogen than *st*Pho1. We have to note that glucan synthesis in Pho1 does not require a primer^91^ like mammalian GP, which requires glycogenin.^92^ Further support comes from the *st*Pho1ΔL78 structure, which revealed that L78 might obstruct the entrance to the channel leading from the active site to the surface of the enzyme.
